# Genetic Diversity and Combining Ability of White Maize Inbred Lines under Different Plant Densities

**DOI:** 10.3390/plants9091140

**Published:** 2020-09-03

**Authors:** Mohamed M. Kamara, Medhat Rehan, Khaled M. Ibrahim, Abdullah S. Alsohim, Mohsen M. Elsharkawy, Ahmed M. S. Kheir, Emad M. Hafez, Mohamed A. El-Esawi

**Affiliations:** 1 Department of Agronomy, Faculty of Agriculture, Kafrelsheikh University, Kafr El-Sheikh 33516, Egypt; mohamed.kamara@agr.kfs.edu.eg (M.M.K.); emadhafez2012@agr.kfs.edu.eg (E.M.H.); 2 Department of Genetics, Faculty of Agriculture, Kafrelsheikh University, Kafr El-Sheikh 33516, Egypt; medhat.rehan@agr.kfs.edu.eg; 3 Department of Plant Production and Protection, College of Agriculture and Veterinary Medicine, Qassim University, Burydah 51452, Saudi Arabia; a.alsohim@qu.edu.sa; 4 Agronomy Department, Faculty of Agriculture, New Valley University, El-Kharga 72511, Egypt; kh_ibrahim75@aun.edu.eg; 5 Department of Agricultural Botany, Faculty of Agriculture, Kafrelsheikh University, Kafr Elsheikh 33516, Egypt; mohsen.abdelrahman@agr.kfs.edu.eg; 6 Soils, Water and Environment Research Institute, Agricultural Research Center, Giza 12112, Egypt; ahmed.kheir@arc.sci.eg; 7 Botany Department, Faculty of Science, Tanta University, Tanta 31527, Egypt

**Keywords:** maize, density tolerance, combining ability, gene effects, genetic diversity

## Abstract

Knowledge of combining ability and genetic diversity are important prerequisites for the development of outstanding hybrids that are tolerant to high plant density. This work was carried out to assess general combining ability (GCA) and specific combining ability (SCA), identify promising hybrids, estimate genetic diversity among the inbred lines and correlate genetic distance to hybrid performance and SCA across different plant densities. A total of 28 F_1_ hybrids obtained by crossing eight adverse inbred lines (four local and four exotic) were evaluated under three plant densities 59,500 (D1), 71,400 (D2) and 83,300 (D3) plants ha^−1^ using spilt plot design with three replications at two locations during 2018 season. Increasing plant density from D1 to D3 significantly decreased leaf angle (LANG), chlorophyll content (CHLC), all ear characteristics and grain yield per plant (GYPP). Contrarily, days to silking (DTS), anthesis–silking interval (ASI), plant height (PLHT), ear height (EHT), and grain yield per hectare (GYPH) were significantly increased. Both additive and non-additive gene actions were involved in the inheritance of all the evaluated traits, but additive gene action was predominant for most traits. Inbred lines L_1_, L_2_, and L_5_ were the best general combiners for increasing grain yield and other desirable traits across research environments. Two hybrids L_2_ × L_5_ and L_2_ × L_8_ were found to be good specific combiners for ASI, LANG, GYPP and GYPH. Furthermore, these hybrids are ideal for further testing and promotion for commercialization under high plant density. Genetic distance (GD) among pairs of inbred lines ranged from 0.31 to 0.78, with an average of 0.61. Clustering based on molecular GD has effectively grouped the inbred lines according to their origin. No significant correlation was found between GD and both hybrid performance and SCA for grain yield and other traits and proved to be of no predictive value. Nevertheless, SCA could be used to predict the hybrid performance across all plant densities. Overall, this work presents useful information regarding the inheritance of maize grain yield and other important traits under high plant density.

## 1. Introduction

Maize (*Zea mays* L.) is one of the main economic crops that subsidize global food security. It is widely used for food, animal feed, edible oil and fuel worldwide [1]. In Egypt, maize is considered the second most important crop, with the annual production of the grain reaching about 7.30 Mt from approximately 0.94 Mha in 2018 [2]. This production is insufficient to meet the demands of a fast-growing population. The gap between production and consumption is approximately 45% [3]. This gap could be narrowed by further increase in the hybrids yield potential and total yield production from unit area. [4]. Increasing planting density is required to increase grain yield production in maize [5]. The average density of intense maize cultivation in the USA is 97,500 plants ha^−1^ [6]. The recommended planting density in Egypt is 53,533 plants ha^−1^ [7], which is around half the amount used in the USA. The use of lower plant densities decreases light interception, leading to high grain production per plant but low grain production per unit area [8]. The yield production could be maximized by growing maize hybrids that can tolerate high plant density up to 100,000 plants ha^−1^. However, high plant densities enhance interplant competition for light, nutrients, and water [9]. Additionally, it increases the anthesis–silking interval [10], thereby increasing kernel abortion [11] and reducing single plant yield. Al-Naggar et al. [12] showed that with increased planting density, plant and ear heights increased, whereas chlorophyll content, grains per ear and thousand grain weights decreased. The tolerance of the current Egyptian maize hybrids to high plant densities is low. This probably attributed to their tallness, decumbent leaf, one-eared and large size [7,13]. Conversely, modern maize hybrids in developed countries are characterized by early silking, short anthesis–silking interval and prolificacy, which are essential adaptive traits to high plant density tolerance [10,14,15,16].

Breeding programs should be directed towards the development of hybrids that are not only high yielding, but also show enhanced adaptability to high plant density tolerance. The successful identification of desirable hybrid combinations depends on the combining ability of the parents and the gene effects involved in the expression of target trait [17]. Furthermore, knowledge of gene action is important to devise an appropriate breeding strategy [18]. General combining ability (GCA) and specific combining ability (SCA) are widely used in selection of good parents and hybrids, respectively [19]. Among different biometrical approaches, the diallel mating design is commonly used by maize breeders to estimate GCA and SCA effects [20,21,22]. GCA is associated with additive gene effects, whereas SCA is typically associated with non-additive gene effects [23]. Both additive and non-additive gene actions were reported to be important in the inheritance of maize grain yield under high plant density [24]. However, the grain yield and other assessed traits under different plant densities among selected maize inbred lines were mostly controlled by additive gene action [7,25].

The assessment of the diversity and genetic distance in the available maize inbreds is important for a hybrid breeding program, in order to identify inbreds that would produce crosses with good levels of heterosis without testing all hybrids combinations [26,27]. Different types of DNA markers are available to estimate genetic distance. The simple sequence repeat (SSR) markers or microsatellites have been considered as the markers of choice owing to their co-dominant, high polymorphic, multi-allelic nature and high reproducibility [28,29,30]. However, contradictory results have been reported with respect to the relationship between genetic distance and hybrid performance in maize. Significant correlations were reported between molecular marker-based GD and F_1_ hybrid grain yield in maize [31,32]. Whereas, other studies reported no significant correlation [33,34]. The objectives of this study were (i) to estimate GCA of the inbred lines and SCA of the hybrids under different plant densities; (ii) to determine the mode of gene action controlling grain yield and other important agronomic traits; (iii) to identify promising hybrids that yield well at high plant density; and (iv) to assess genetic diversity among the eight inbred lines and correlate genetic distance to hybrid performance and SCA.

## 2. Results

### 2.1. Analysis of Variance

The analysis of variance (ANOVA) revealed highly significant mean squares for locations (L), densities (D), hybrids (H) and their interactions (L × D, H × L, H × D and H × D × L) for all the studied characteristics (Table 1). Moreover, general combining ability (GCA) and specific combining ability (SCA) mean squares were highly significant for all the measured traits. The magnitude of GCA mean squares was higher than that of SCA mean squares (the ratio of GCA/SCA was higher than the unity) for all the studied traits, except number of kernels per row (NKPR) trait. Significant mean squares of GCA × L, SCA × L, GCA × D, SCA × D, GCA × L × D, SCA × L × D interactions were detected for all the studied traits, except GCA × L and GCA × L × D for leaf angle (LANG) and chlorophyll content (CHLC), GCA × D for ear diameter (ED) and SCA × D for EHT, LANG and ED were not significant.

### 2.2. Changes in the Studied Traits Due to Increased Plant Density

**Figure 1:**
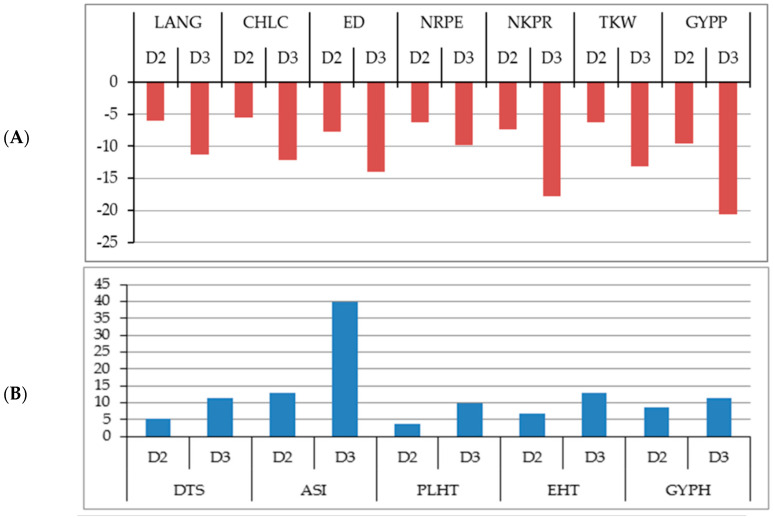
Shows the changes due to increased plant density: (**A**) reduction in leaf angle (LANG), chlorophyll content (CHLC), ear diameter (ED), number of rows per ear (NRPE), number of kernels per row (NKPR), thousand kernel weight (TKW) and grain yield per plant (GYPP); (**B**) increase in days to 50% silking (DTS), anthesis–silking interval (ASI), plant height (PLHT), ear height (EHT) and grain yield per hectare (GYPH) under D2 and D3 in compared with D1.

Across the two locations, the mean of grain yield per plant (GYPP) was significantly decreased as plant density increased from D1 to D2 and D3 by −9.60 and −20.59%, respectively, as compared to D1 (Figure 1A). This reduction was accompanied by reductions in leaf angle (LANG) (−5.97 and −11.23%), chlorophyll content (CHLC) (−5.48 and −12.15%) and all yield attributes; ear diameter (ED) (−7.68 and −14.01%), number of rows per ear (NRPE) (−6.21 and −9.83%), number of kernels per row (NKPR) (−7.38 and −17.77%), and thousand kernel weight (TKW) (−6.39 and −13.13%) at plant density of D2 and D3, respectively, as compared to D1. Conversely, high plant density (D2 and D3) caused a significant increase in grain yield per hectare (GYPH) compared with the low density (D1) by 8.48 and 11.23%, respectively (Figure 1B). Similarly, D2 and D3 caused significant increases in days to 50% silking (DTS) (5.10 and 11.31%), anthesis–silking interval (ASI) (12.87 and 39.88%), plant height (PLHT) (3.78 and 9.75%) and ear height (EHT) (6.64 and 12.86%) as compared with low plant density (D1), respectively.

### 2.3. Performance of F_1_ Hybrids

The mean performances of the 28 F_1_ hybrids and the commercial check hybrid SC128 for all the studied characteristics are provided in Supplementary Materials, Table S1. The evaluated hybrids showed a wide variation for all studied traits under all plant densities. The mean values for DTS were 58.22 days in D1, 61.19 days under D2, and 64.80 days in D3 (Table 2). The earliest hybrids were L_1_ × L_3_ at D1, L_3_ × L_4_ at D2 and L_1_ × L_4_ at D3, while the latest hybrids were L_6_ × L_8_ under D1 and D2 and L_3_ × L_6_ under D3 (Table 2). A total of 21, 17 and 4 hybrids were significantly earlier than the check hybrid SC128 under D1, D2 and D3, respectively (Supplementary Materials, Table S1). Likewise, the means of ASI were 3.26 days in D1, 3.68 days under D2, and 4.56 days in D3. The longest ASI was shown by the hybrid L_3_ × L_7_, and the shortest one was shown by L_2_ × L_5_ under the three plant densities (Table 2). The highest PLHT mean was 263.52 cm in D3, while it was 240.122 cm and 249.20 cm in D1 and D2, respectively. The tallest hybrids were L_4_ × L_7_ under D1 and D3, and L_2_ × L_4_ under D2, while the shortest hybrid was L_2_ × L_6_ under the three plant densities (Table 2). The means of the EHT were 117.86, 125.68 and 133.02 in D1, D2 and D3, respectively. A total of 12, 11 and 14 hybrids were significantly shorter than the check hybrid SC128 under D1, D2 and D3, respectively (Supplementary Materials, Table S1).

The hybrid L_6_ × L_7_ had the highest ear height under the three plant densities, while the hybrids L_3_ × L_6_ in D1 and L_2_ × L_6_ under D2 and D3 had the lowest ear heights (Table 2). A total of 13, 20 and 19 hybrids had significantly lower ear placement than the check hybrid SC128 under D1, D2 and D3, respectively (Supplementary Materials, Table S1). Furthermore, the hybrid L_4_ × L_5_ displayed the lowest LANG, while L_3_ × L_7_ gave the highest one under the three plant densities. The means of CHLC were 50.34, 47.59 and 44.23 SPAD units under D1, D2 and D3, respectively. The highest hybrid in CHLC was L_2_ × L_8_, while the lowest hybrid was L_7_ × L_8_ across the three plant densities (Table 2). Moreover, the hybrids L_5_ × L_6_ at D1, L_3_ × L_4_ at D2 and L_1_ × L_5_ at D3 significantly surpassed the check hybrid SC128 for this trait (Table S1). The means of ED were 5.16 cm in D1, 4.76 cm under D2, and 4.44 cm in D3. The hybrid L_1_ × L_7_ at D1 and L_2_ × L_4_ at D2 and D3 exhibited the lowest ED, while L_1_ × L_8_, L_1_ × L_3_ and L_1_ × L_4_ gave the highest ones under D1, D2 and D3, respectively (Table 2). The mean for the NRPE was 14.83 in D1 and 13.91 in D2, while it was 13.37 in D3. The hybrid L_2_ × L_5_ under D1 and L_1_ × L_5_ under D2 and D3 exhibited the highest NRPE, while L_3_ × L_7_ in D1, L_3_ × L_4_ under D2 and L_1_ × L_3_ in D3 had the lowest mean values (Table 2). Additionally, two hybrids under D1, four hybrids at D2 and three hybrids at D3 possessed higher NRPE than the check hybrid SC128 (Supplementary Materials, Table S1). The mean values of the NKPR were 40.28, 37.31 and 33.12 for D1, D2 and D3, respectively. The hybrid L_2_ × L_8_ had the highest NKPR, but the hybrid L_1_ × L_5_ displayed the lowest one under the three plant densities. Means of the TKW were 356.0 g, 333.24 g, and 309.26 g in D1, D2, and D3, respectively. The heaviest TKW was assigned for the hybrids L_2_ × L_8_ under D1 and L_1_ × L_4_ under D2 and D3, whereas the hybrids L_3_ × L_8_ in D1, L_5_ × L_6_ under D2 and L_5_ × L_7_ under D3 exhibited the lightest TKW (Table 2). Furthermore, four hybrids under D1, five hybrids at D2 and three hybrids at D3 significantly exceeded the check hybrid SC128 for this trait (Supplementary Materials, Table S1). The highest mean of GYPP was 170.11 g in D1, while it was 153.78 and 135.09 g in D2 and D3, respectively. Conversely, the highest mean of GYPH was obtained in D3 (11.26 t ha^−1^), followed by D2 (10.98 t ha^−1^) and then by D3 (10.12 t ha^−1^) (Table 2). The hybrid L_2_ × L_8_ was the top yielding hybrid and significantly out-yielded the check hybrid SC128 by 9.98, 13.16 and 10.26% under D1, D2 and D3, respectively. Moreover, the hybrid L_2_ × L_5_ significantly surpassed the check hybrid SC128 by 5.26% only under D2 (Supplementary Materials, Table S1). The optimum plant density for obtaining the highest GYPH was D3 for all hybrids, except the hybrids; L_2_ × L_7,_ L_3_ × L_4,_ L_3_ × L_7_ and L_2_ × L_8_, where the optimum density was D2 (Supplementary Materials, Table S1). This indicates that the optimum plant density is genotype dependent and should be identified separately for each hybrid.

### 2.4. General Combining Ability (GCA) Effects

Estimates of GCA effects are presented in Table 3. High positive values of GCA effects would be of interest for all studied characteristics in question, except DTS, ASI, PLHT, EHT and LANG where high negative values would be desirable from the breeder point of view. Results showed that the highest significant and negative GCA effects under the three plant densities were obtained by the inbred lines L_1_ and L_3_ for DTS; L_1_, L_2_ and L_5_ for ASI; L_1_, L_5_, L_6_ and L_8_ for PLHT; L_3_, L_5_ and L_8_ for EHT and L_1_, L_2_ and L_4_ for LANG. Additionally, the inbred lines L_4_ in D1 and D2, as well as L_5_ in D3 for DTS; L_4_ in D3 and L_8_ in D1 and D2 for ASI; L_2_ in D3 and L_3_ under D1 and D3 for PLHT; and L_5_ under D1 and D3 for LANG also expressed significant and negative GCA effects for these traits. In contrast, the inbred lines L_1_ in D2 and D3, L_5_ under D1 and L_2_ under the three plant densities possessed significant and positive GCA effects for CHLC. Regarding ED, the inbred lines L_1_ and L_8_ in D1 and D3 as well as L_3_ in D2 had significant and positive GCA effects.

The highest positive and significant GCA effects for NRPE belonged to L_1_ in D2 and D3, L_5_ and L_8_ in D1 and D3, and L_2_ under the three plant densities. Likewise, the inbreds L_3_ and L_7_ in D1; L_1_ and L_6_ in D3 and L2 under the three plant densities were determined and considered to be good general combiners for NKPR. The highest positive and significant GCA effects for TKW belonged to L_1_ and L_2_ under the three plant densities, L_4_ under D1 and D2 and L_6_ under D3. Furthermore, the inbred lines L_1_, L_2_ and L_5_ under the three plant densities and L_8_ under D3 had significant and positive GCA effects for GYPP and GYPH. Based on the summarized results, it can be concluded that parental lines L_1,_ L_2_ and L_5_ had the highest GCA effects for grain yield and the majority of studied traits.

### 2.5. Specific Combining Ability (SCA) Effects

The estimated SCA values under the three plant densities across two locations are presented in Table 4. The hybrids that presented the highest significant and negatives SCA effects (desirable) under the three plant densities were L_1_ × L_6,_ L_2_ × L_4_, L_3_ × L_5,_ L_3_ × L_8_, L_4_ × L_7_ for DTS; L_1_ × L_7,_ L_2_ × L_5,_ L_2_ × L_7,_ L_2_ × L_8,_ L_3_ × L_4,_ L_3_ × L_6_ and L_4_ × L_5_ for ASI; L_1_ × L_4,_ L_2_ × L_6_, L_2_ × L_7,_ L_2_ × L_8,_ L_3_ × L_4_ and L_3_ × L_7_ for PLHT; L_1_ × L_7,_ L_1_ × L_8,_ L_2_ × L_6_ and L_3_ × L_6_ for EHT and L_1_ × L_4,_ L_1_ × L_5_, L_1_ × L_6,_ L_1_ × L_7,_ L_2_ × L_5,_ L_2_ × L_8,_ L_3_ × L_4,_ L_3_ × L_6,_ L_4_ × L_5,_ L_4_ × L_7_ and L_7_ × L_8_ for LANG. On the contrary, the hybrid combinations; L_1_ × L_7,_ L_2_ × L_8,_ L_3_ × L_4_ and L_5_ × L_6_ for CHLC; L_2_ × L_5_ and L_2_ × L_7_ for ED; L_1_ × L_5_, L_2_ × L_3,_ L_3_ × L_6_ and L_6_ × L_7_ for NRPE; L_1_ × L_6,_ L_2_ × L_8_ and L_6_ × L_7_ for NKPE; L_1_ × L_4,_ L_1_ × L_6,_ L_2_ × L_5,_ L_2_ × L_8,_ L_3_ × L_5,_ L_4_ × L_5,_ L_6_ × L_7_ and L_7_ × L_8_ for TKW and L_1_ × L_3,_ L_1_ × L_6,_ L_2_ × L_5,_ L_2_ × L_8,_ L_3_ × L_4,_ L_3_ × L_6,_ L_4_ × L_5,_ L_6_ × L_7_ and L_7_ × L_8_ for GYPP and GYPH had the highest significant and positive SCA effects (desirable) under the three plant densities. Moreover, the hybrids L_1_ × L_5_ in D2 and D3, L_4_ × L_7_ in D1 and D2 and L_2_ × L_4_ and L_5_ × L_7_ under D3 displayed significant and positive SCA effects for GYPP and GYPH. It is notable that the crosses that showed high SCA effects for GYPP and GYPH also showed desirable SCA effects for some other traits, i.e., DTS, LANG, NKPE and TKW for the hybrid L_1_ × L_6_; ASI, LANG and TKW for the two hybrids L_2_ × L_5_ and L_4_ × L_5;_ ASI, PLHT, LANG, CHLC, NKPR and TKW for the hybrid L_2_ × L_8_ and PLHT, NRPE, NKPR and TKW for the hybrid L_6_ × L_7_.

### 2.6. SSR Polymorphisms, Genetic Distance (GD) and Cluster Analysis

**Figure 2:**
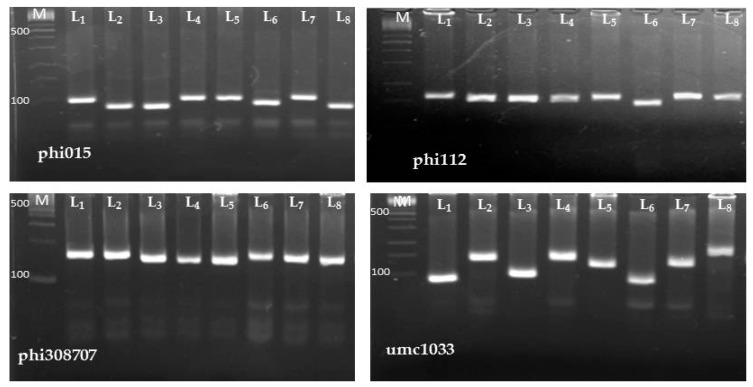
Amplification pattern of representative SSR markers with the eight maize inbred lines (L_1_–L_8_). M refers to the 100 bp DNA ladder.

**Figure 3:**
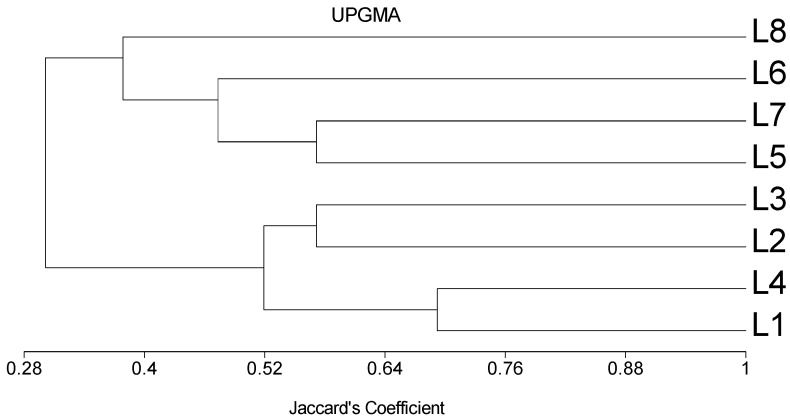
Dendrogram of the eight maize inbred lines constructed from SSR data using (UPGMA) according to Jaccard’s coefficients.

Out of twenty-two SSR primer pairs analyzed, ten were polymorphic among the eight inbreds studied (Table 5). The primer pairs generated a total of 80 polymorphic fragments (Figure 2). The number of alleles per locus ranged from 2 to 6, with an average number of 2.7 alleles/locus (Table 5). The major allele frequency had an average of 0.59 with a range extended from 0.25 to 0.88. The gene diversity and polymorphic information content (PIC) averaged 0.50 and 0.41, with ranges of 0.22–0.81 and 0.19–0.79, respectively. The umc1033 locus showed the highest gene diversity and PIC (Table 5). Genetic distance estimates based on SSR markers ranged from 0.31 to 0.78 with an average of 0.61 (Table 6). The lowest genetic distance (0.31) was obtained between the inbred lines (L_1_ and L_4_), whereas the highest genetic distance (0.78) was observed between the inbred lines (L_1_ and L_8_), (L_2_ and L_5_), (L_2_ and L_6_) and (L_2_ and L_8_). The dendrogram constructed based on GD revealed two main clusters; L_1_, L_2_, L_3_ and L_4_ constituted the first group, while L_5_, L_6_, L_7_ and L_8_ formed the second one (Figure 3).

### 2.7. Association between Genetic Distance, F_1_ Hybrid Performance and SCA

**Figure 4:**
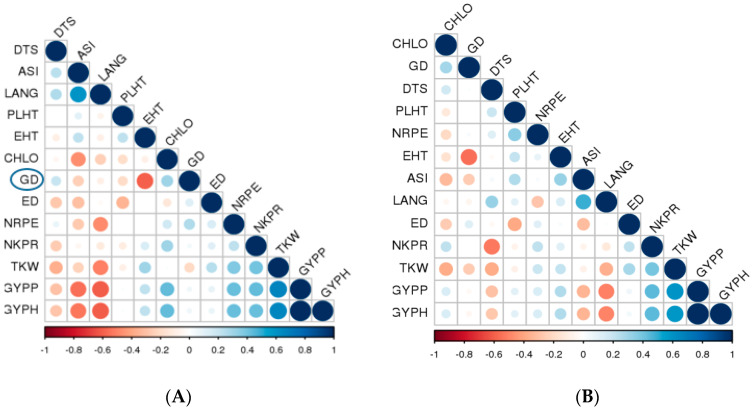
Corrplot depicting correlation coefficient of genetic distance based on molecular data with F_1_ hybrid performance (**A**) and SCA (**B**) for all studied traits. GD: genetic distance, DTS: days to 50% silking, ASI: anthesis–silking interval, PLHT: plant height, EHT: ear height, LANG: leaf angle, CHLC: chlorophyll content, ED: ear diameter, NRPE: number of rows per ear, NKPR: number of kernels per row, TKW: thousand kernel weight, GYPP: grain yield per plant and GYPH: grain yield per hectare.

Correlations between GD estimated for pairs of inbred lines with each of F_1_ hybrid performance and SCA were not significant for all measured traits (Table 7, Figure 4A,B). However, significant and positive association was observed between F_1_ hybrid performance and SCA for all the studied traits across the three plant densities (Table 7).

## 3. Discussion

### 3.1. Analysis of Variance and Hybrid Performance

The significant mean squares of L, D and H observed for all the studied characteristics (Table 2), indicate that the tested locations and densities were dissimilar and there were adequate genetic differences among the hybrids for effective selection of all the studied traits. Significant differences among maize hybrids under different plant densities were also reported [10,35,36,37]. The presence of significant mean squares for H × D interaction, indicated inconsistent performance of the hybrids across plant densities. In that context, the ranks of maize hybrids differed from one density to another for all measured traits. Therefore, selection of hybrids under various plant densities may be a promising strategy to improve the adaptation of maize hybrids to higher plant density. These results are consistent with the findings of other studies [12,13,36,38].

The significant GCA and SCA effects imply that both additive and non-additive gene effects are involved in governing all traits. The inheritance of a specific trait could be identified based on the ratio of GCA/SCA variances. In the present study, the GCA/SCA ratio was greater than unity for all evaluated characteristics, except NKPR, which indicated the preponderance of additive gene effects in controlling the inheritance of all measured traits, except NKPR which was mainly controlled by non-additive gene action. Therefore, selection breeding methods can be effective for improvement of these traits. This finding is in agreement with that of Mason and Zuber [25] and Al-Naggar et al. [7], who reported that additive genetic effects were important in the inheritance of grain yield and other agronomic traits under different plant densities. However, this result is in contrast to the findings of other studies [36,39], who reported that non-additive gene effects were found to be more important in controlling grain yield inheritance under varying plant densities.

The significant GCA × L and GCA × D interactions mean squares for most traits in the present study indicate that the GCA effects of the inbred lines varied significantly under different environments. This result is in agreement with the findings of several authors [17,26,40,41]. Likewise, the significant SCA × L and SCA × D interactions observed for most traits implied that the performance of the hybrids was not consistent under varying research environments. This suggests the need for extensive evaluation of the hybrids in multiple environments in order to identify high yielding and most stable hybrids tolerant to high plant densities [39].

The highest GYPP of all evaluated hybrids in this study was observed under low density (D1), where competition between plants is minimum [12]. As planting density increases, resources to each plant (water, nutrients and light interception) decrease, increasing plant–plant competition and in turn reducing the assimilated supply to developing cobs and, consequently, resulting in a reduction in grain yield per plant [42,43,44]. The observed reduction in GYPP due to elevating plant density from D1 to D2 and D3 in this study could be a result of the reduction in all yield attributesED, NRPE, NKPR and TKW. These results are consistent with Tang et al. [45], who stated that increasing plant density in maize leads to a reduction in ear diameter, grains per ear, thousand kernels weight and finally single plant yield. Hashemi et al. [46] also demonstrated that grain yield per plant and all yield components linearly decreased with increasing plant density. Moreover, increasing plant density also reduced LANG and CHLC. The decrease in the leaf angle and chlorophyll content in response to high plant density has also been reported previously in maize [13,47,48].

On the other hand, high plant density (D3) caused significant increases in DTS, ASI, PLHT, EHT and GYPH compared with the low density (D1). Delayed silking and increased ASI period, as symptoms of intense interplant competition for growth resources, can be associated with significant yield reductions [15,49]. Increasing plant density initiated greater stress during pollination that can lead to increasing kernel abortions and decreasing grain fill [8,11]. These two traits (early DTS and short ASI) could be effective indicators for selecting high density tolerance hybrids [50]. The increased values of PLHT and EHT might be related to the stress imposed on maize plants due to competition for light resulting from elevated plant density which potentially increase stem elongation [51,52]. The increase in GYPH with increasing plant density is largely attributed to the higher number of plants per unit area. This suggested that the increase in GYPH due to increased plant density may offset the reduction in GYPP due to competition between plants. These results are in accordance with the results reported in other studies [10,12,53,54].

The two hybrids L_2_ × L_5_ and L_2_ × L_8_ had the highest GYPP and GYPH under three plant densities, and could be considered the most highly responsive and tolerant to high plant density. Interestingly, the hybrid L_2_ × L_8_ significantly outyielded the check hybrid SC128 under all densities; moreover, it had outstanding features, such as short ASI, short plant and ear position, erect leaf under high plant density. Therefore, this hybrid should be tested extensively in multilocation trials and promoted for adoption to high plant density tolerance. Similar to our results, Al-Naggar et al. [12] reported that the selection of hybrids with high grain yield, better plant and ear heights, short ASI, and erect leaf under high plant density stress is important for the development of tolerant hybrids to high plant densities.

### 3.2. GCA and SCA Estimates

Combining ability analysis helps in the identification of parents with good GCA effects and hybrids with good SCA effects [23]. Selection of parents giving good-performing hybrids is one of the challenges facing breeders. Parents with desirable GCA effect for the target traits can be used to accumulate favorable alleles by recombination and selection [55]. In the current study, high GCA values for the evaluated traits were scattered among the eight inbred lines and changed across plant densities, demonstrating the effects of plant densities on GCA values. Moreover, none of the inbred lines exhibited significant GCA effects for all the measured traits under any of the testing densities. Similar results were reported by other researchers [56,57]. The significant and negative GCA effects were displayed by the inbreds L_1_ and L_3_ for DTS and L_1_, L_2_ and L_5_ for ASI across the three plant densities, indicating that, these inbreds could be good combiners and possessed favorable alleles towards earliness. Likewise, inbred lines L_5_ and L_8_ were the best general combiners for reduced plant and ear heights which are important for lodging tolerance especially under high plant density. The inbred line L_2_ had the highest positive GCA values for CHLC, NRPE, NKPR and TKW suggesting that this line could be good combiner for improving these traits. Moreover, the best general combiners for GYPP and GYPH were L_1_, L_2_, and L_5_ under the three plant densities and L_8_ under D3. These inbreds could transfer desirable alleles for improved grain yield to their progenies to develop hybrids tolerant to high plant density. The superiority of these inbreds in GCA effects for grain yield was associated with their superiority in GCA effects for some other traits. Interestingly, the inbred line L_1_, which had desirable GCA effects for GYPP and GYPH, was also found to be good a general combiner for earliness, short ASI, short PLHT, reduced LANG and increased TKW. Previous findings proved that positive GCA effects for grain yield and negative GCA effects for DTS, PLHT, and LANG traits are a good indicator of high plant density tolerance [13]. Thus, the inbred line L_1_ has potential to be used to improve maize grain yield under high plant density.

Estimates of SCA effects provide important information about the non-additive gene effects (dominance and epistatic interaction), which can also be related to hybrid vigor, assisting in the selection of the best hybrid combinations [58]. The highly positive and significant SCA effects for grain yield and its components indicated that the produced hybrids were good specific combiners for developing high-yielding hybrids [1]. In the present study, the most promising specific combiners for grain yield (GYPP or GYPH) and some of its components were L_1_ × L_3_, L_1_ × L_6_, L_2_ × L_5_, L_2_ × L_8_, L_4_ × L_5_ and L_7_ × L_8_ under the three plant densities. These hybrids involved at least one high GCA parent, which could be exploited by conventional breeding procedures. This finding is in line with the result reported in other studies [56,59]. In their studies, high SCA was observed in cross combinations involving one line with high GCA and another with low GCA effects.

Two hybrids, L_2_ × L_5_ and L_2_ × L_8_, had desirable significant positive SCA coupled with high mean grain yield under the three plant densities, revealing good correspondence between mean grain yield and SCA effects [1]. Regardless of their significant SCA effects, three crosses L_3_ × L_4,_ L_3_ × L_6_ and L_6_ × L_7_, constituted from parents with low × low GCA effects for GYPP and GYPH were not favorable due to insufficient additive variance. This indicates that both GCA and SCA should be taken into consideration in the selection of elite parents for the development of heterotic hybrids [18]. It is notable that none of the hybrids exhibited significant SCA effects for all the traits. However, the hybrids L_2_ × L_5_, L_2_ × L_8_ and L_4_ × L_5_ were found to be good specific combiners for more than one trait, such as ASI, LANG, TKW, GYPP and GYPH. Accordingly, these hybrids would be useful to increase maize grain yield under high plant density for their complementary characteristics, including, short ASI, erect leaf and high grain yield under high plant density. In concordance with the findings reported here, desirable significant SCA under high plant density for ASI, LANG and grain yield has previously been reported by Al-Naggar et al. [13].

### 3.3. SSR Polymorphisms, Genetic Distance (GD) and Cluster Analysis

The mean number of alleles (2.7) per locus obtained in this study was close to the values reported by other researchers [26,27,34], who detected averages of 2.9, 2.57 and 3.0 alleles per locus, respectively. However, it was lower than the 6.21 alleles/locus reported by Oppong et al. [60] or the 5.7 alleles/locus found by Oyekunle et al. [61] in maize inbred lines using SSR markers. The differences in the means of alleles among different studies could be attributed to the differences in sample size, repeat length and number of the SSR markers involved in the studies [27]. The lower values observed in this study could arise from the small number of lines used for genotyping.

The PIC demonstrates the informativeness of the SSR loci and their potential to detect differences among the inbred lines based on their genetic relationships [62]. Informative markers can be categorized as highly informative (PIC > 0.5), reasonably informative (0.5 < PIC < 0.25) and slightly informative (PIC < 0.25), as reported by Botstein et al. [63]. Accordingly, four markersumc1014, phi112, phi015 and umc1033 with high PIC values, and hence high discriminatory power, were identified. The average gene diversity (0.50) detected among the tested inbred lines in this study indicated high levels of polymorphisms within the inbred lines. This result is in close agreement with the findings reported in other studies [30,64]. The frequency of the most common (major) alleles had an average of 0.59, suggesting that 59.0% of the studied inbreds shared a common major allele at any of the tested loci.

Assessing the genetic diversity is essential for enhancing the yield and conservation strategies of main crops [65,66,67,68,69,70], such as maize that has high an economic importance [71]. The average genetic diversity existing among all the inbred lines was relatively high (0.61). This indicated that there was considerable genetic diversity among the inbreds based on the microsatellite markers analysis [72]. The largest GD in this study was between the Egyptian (local) and CIMMYT (exotic) inbred lines. The relatively large genetic distance between local and exotic lines, suggesting the opportunity to use these lines for the development of high-yielding and stress-tolerant hybrids. Indeed, the two high-yielding hybrids (L_2_ × L_5_ and L_2_ × L_8_) under the three plant densities consisted of local × exotic line combinations. This indicates that novel and complementary alleles existing in the germplasm from the two countries can be exploited for superior maize hybrid development and population improvement [73]. Moreover, it implies the potential benefits of exchanging germplasm between breeding programs for the development of high yielding and density tolerant hybrids.

The dendrogram constructed using the UPGMA clustering grouped the inbred lines into two main clusters, which generally agreed with their origin. One cluster was composed of CIMMYT inbred lines, while the other consisted of local inbreds. This result is consistent with the findings of Mageto et al. [17], who reported that clustering based on GD grouped maize inbred lines according to their origin. Similarly, [34,64] revealed the effectiveness of SSR markers for classifying maize inbreds according to their origin in their studies.

### 3.4. Association between Genetic Distance, F1 Hybrid Performance and SCA

Our results showed that GD of the parental inbreds was not significantly correlated with the mean of F_1_ hybrids for any of the evaluated traits across the tested environments. This implied that the SSR-based GD could not be used to predict the performance of F_1_ hybrids in this study. This result is consistent with those reported by [26,33,34,40]. Bernardo [74] attributed this poor correlation to the lack of linkage between genes controlling the trait and markers used to estimate GD, inadequate genome coverage and different levels of dominance among hybrids. Contrary to the current finding, a significant correlation was reported between molecular GD and F_1_ hybrid performance [32,75]. There was no significant correlation between GD and SCA for all the traits, suggesting that SSR-based GD might not be effective in predicting SCA effects in the studied materials. Similarly, non-significant association between genetic distances and SCA was reported by [40,76]. However, Betran et al. [75] reported a significant correlation between GD and SCA for maize grain yield. Furthermore, our results showed that SCA effects were significantly correlated with F_1_ hybrid performance for all the traits. This indicated that SCA could be used to predict the performance of F_1_ hybrids. This result is in agreement with the findings of [17,26].

## 4. Conclusions

This study revealed a considerable variability among F_1_ hybrids for all traits under different plant densities. Additive and non-additive gene effects are involved in the genetic control of all traits, with a predominance of the additive gene action for most traits. Selection of potential hybrids for density tolerance breeding programs should be based on both GCA and SCA effects. The inbred lines L_1_ and L_3_ were identified as excellent combiners for earliness, L_5_ and L_8_ for reduced plant and ear heights and L_1_, L_2_, and L_5_ for increased grain yield under varying plant densities. The best hybrids L_2_ × L_5_ and L_2_ × L_8_ for grain yield and other multiple traits were identified for further evaluation. The estimated GD based on SSR markers in this study could not be used to predict the hybrids performance and SCA effects. Nevertheless, SCA could be used to predict the hybrids performance across all plant densities. Although SSR determined that GD was not useful in predicting hybrid performance and SCA effects, it was effective in classifying the inbred lines according to their origin, signifying the efficiency of SSR marker for diversity and clustering analyses. The findings of the present study might have important implications for breeding programs designed to improve density tolerance in maize.

## 5. Materials and Methods

### 5.1. Plant Materials

Eight white maize (*Zea mays* L.) inbred lines showing clear differences in grain yield and other agronomic characteristics were chosen as parents in this study. Four inbreds (L_1_, L_2_ L_3_ and L_4_) were obtained from Maize Research Department, Agricultural Research Center (ARC) in Egypt and the other four (L_5_, L_6_, L_7_ and L_8_) were introduced from the International Maize and Wheat Improvement Center (CIMMYT). The parental codes, names and sources of these inbred lines are listed in Table 8.

### 5.2. Production and Evaluation of F_1_ Hybrids

In the 2017 season, all possible diallel crosses (excluding reciprocals) were made among the eight inbred lines to obtain seeds of 28 F_1_ hybrids. In the 2018 season, the resulting 28 F_1_ white hybrids plus the commercial check hybrid SC128 were evaluated under three plant densities, i.e., 59,500 (D1), 71,400 (D2) and 83,300 (D3) plants ha^−1^ at two locations. The two locations were El-Mahmoudia, El-Behira, Egypt ((31°3′ N, 30°48′ E)) in a private farm, and the Experimental Farm, Faculty of Agriculture, Kafrelsheikh University, Egypt ((31°6′ N, 30°56′ E)). A split-plot design in randomized complete blocks (RCB) arrangement with three replications was used in each location. The three plant densities were located at the main plots, while the hybrids were located at the sub plots. Each subplot consisted of one ridge of 6 m long and 0.7 m width. Two seeds were sown in hills at 24, 20 and 17 cm apart, and thereafter (before the 1st irrigation) were thinned to one plant/hill to achieve the three plant densities, i.e., D1, D2 and D3, respectively. Phosphorus at the rate of 476 kg ha^−1^ in the form of calcium super phosphate (15.5% P_2_O_5_) was added to the soil during seedbed preparation, and potassium sulphate (48% K_2_O) at a level of 120 kg ha^−1^ was applied after thinning. Moreover, nitrogen at the rate of 286 kg ha^−1^ was added in two equal doses before the first and second irrigation. All other standard agronomic practices including weed control were followed in each location. Soil analysis was conducted on soil samples collected from 30 cm depth from each location according to Association of Officinal Analytical Chemists (A.O.A.C 2005) [77] (Supplementary Materials, Table S2). Additionally, the meteorological data are presented in the Supplementary Materials, Figure S1.

### 5.3. Data Collection

Data were collected on days to 50% silking (DTS, days from the planting to 50% extrusion of silks from the plants), anthesis–silking interval (ASI, calculated as the difference between days to 50% silking and days to 50% anthesis), plant height (PLHT, measured in cm as the distance from the soil surface to the top of the first tassel branch) and ear height (EHT, measured in cm as the distance from the soil surface to the base of the topmost ear). Leaf angle (LANG) (°) was measured as the angle between stem and blade of the leaf just above ear leaf. Chlorophyll content (CHLC, SPAD units) was measured by hand-held chlorophyll meter (SPAD-502; Minolta Sensing Co., Ltd., Hangzhou, Japan) from the leaf of the top-most ear. The LANG and CHLC values were recorded on ten guarded plants within each plot, and then the values were averaged per plot. At harvest, ear diameter (ED), number of rows per ear (NKPR), number of kernels per row (NKPR), thousand kernel weight (TKW), grain yield per plant (GYPP, in g plant^−1^) and grain yield per hectare (GYPH, in ton ha^−1^) were estimated. Plots were hand-harvested, and the weight of the shelled grain (adjusted to 15.5% grain moisture content) was used to calculate GYPP and GYPH. Grain moisture at harvest was measured using a hand-held moisture meter.

### 5.4. Molecular Analysis

#### 5.4.1. DNA Isolation

Leaves were sampled from 10 to 15 seedlings of each inbred line after twenty days from planting. Genomic DNA was isolated using CTAB method [78]. DNA quantity as well as quality was assessed using NanoDrop spectrophotometer (ND-1000, USA).

#### 5.4.2. SSR Primers and PCR Amplification

Twenty-two SSR markers were randomly selected from the MaizeGDB database (www.maizegdb.org). The 22 primer pairs were tested to identify the polymorphic ones. Only ten markers were found to be polymorphic and they used for the SSR analysis (Supplementary Materials, Table S3). PCR was performed in a volume of 10 μL reaction mixture containing 1 μL of 20 ng/μL genomic DNA template, 1 unit Taq DNA polymerase (Promega, Madison, WI, USA), 2 mM MgCl2, 0.2 mM dNTPs and 0.5 μM of reverse and forward primer. The PCR reaction was initially started by denaturation at 94 °C for 2 min, followed by 35 cycles consisting of denaturation at 94 °C for 30 sec, 30 sec of annealing at 55 °C, 30 sec of extension at 72 °C and a final extension of 3 min at 72 °C. Amplified products were electrophoresed on 1.5% agarose gel. The gels were stained with ethidium bromide and then distained with tap water and photographed using gel documentation system (UVITEC, Cambridge, UK).

### 5.5. Statistical Analysis

Analysis of variance (ANOVA) was computed for all data using SAS software (SAS Institute Inc, 2008). Combined analysis of variance of the split-plot design across the two locations was performed if the homogeneity test was non-significant. Least significant difference (LSD) values were calculated to test the significance of differences between means according to Steel et al. [79]. General combining ability (GCA) effects of the parents and specific combining ability (SCA) effects of the hybrids as well as their mean squares were computed according to Griffing’s method 4 model I [80], using the DIALLEL-SAS program [81]. The testing of significance of GCA and SCA effects was done at 5% and 1% probability. Pearson’s coefficients of correlation (r) were calculated and plotted using the package corrplot [82]. Based on the mean of each trait the reduction or increase due to increased plant density was calculated as follow:

Change% = 100(D2 or D3 − D1)/D1

### 5.6. SSR Data Analysis

The amplified bands were scored for each SSR marker based on the presence or absence of bands, generating a binary data matrix of (1) and (0) for each marker. The number of alleles per locus, major allele frequency, gene diversity and polymorphic information content (PIC) were calculated to assess allele diversity of each marker. The value of polymorphic information content (PIC) of each SSR marker was determined as described by Botstein et al. [63] as follows:

$$1-\sum_{i=1}^{n}P_{j}^{2}-\sum_{i=1}^{n-1}\sum_{j=i+1}^{n}2P_{i}^{2}P_{j}^{2}$$

where P*_i_* and P*_j_* are the frequencies of the *i*th and *j*th allele of a given marker, respectively.

Genetic distances between pairs of inbred lines were calculated according to [83], using the PAST program. The dendrogram tree was generated with the unweighted pair group method using arithmetic averages (UPGMA) by the computational package MVSP version 3.1.

## Figures and Tables

**Table 1:** Analysis of variance for the evaluated crosses under three plant densities combined across two locations for all the studied traits.

**SOV**	**DF**	**DTS**	**ASI**	**PLHT**	**EHT**	**LANG**	**CHLC**
Locations (L)	1	1114.26 **	12.87 **	16,592.96 **	6489.21 **	108.64 *	400.29 **
Rep (L)	4	15.14	0.58	325.06	138.65	14.87	10.84
Densities (D)	2	1899.48 **	73.14 **	23,422.30 **	9384.04 **	603.65 **	1585.67 **
L × D	2	308.23 **	22.39 **	9852.25 **	5708.38 **	27.17 **	180.79 **
Error a	8	1.07	0.19	121.77	53.15	3.33	2.34
Hybrids (H)	27	28.04 **	7.89 **	6842.02 **	2056.81 **	425.82 **	119.32 **
GCA	7	57.16 **	12.03 **	11,397.27 **	2447.44 **	836.14 **	162.00 **
SCA	20	17.85 **	6.44 **	5247.69 **	1920.08 **	282.20 **	104.38 **
H × L	27	61.66 **	0.83 **	796.40 **	362.93 **	4.15 **	3.61 **
GCA × L	7	52.48 **	0.88 **	915.53 **	309.35 **	3.16	3.57
SCA × L	20	64.88 **	0.81 **	754.71 **	381.68 **	4.49 **	3.62 *
H × D	54	5.01 **	0.31 **	254.88 **	60.51 **	4.57 **	10.19 **
GCA × D	14	4.54 **	0.30 **	212.94 **	78.99 *	6.63 **	19.13 **
SCA × D	40	5.18 **	0.31 **	269.56 **	54.03	3.85	7.06 **
H × D × L	54	63.25 **	0.88 **	592.24 **	397.54 **	4.11 *	4.30 **
GCA × L × D	14	64.03 **	0.86 **	544.25 **	363.40 **	3.90	2.64
SCA × L × D	40	62.97 **	0.88 **	609.03 **	409.48 **	4.19 *	4.88 **
Error b	324	0.81	0.14	84.07	42.33	2.69	1.93
GCA/SCA		3.20	1.87	2.17	1.27	2.96	1.55
**SOV**	**DF**	**ED**	**NRPE**	**NKPR**	**TKW**	**GYPP**	**GYPH**
Locations (L)	1	2.09 *	36.35 **	353.21 **	8232.88 **	12,079.40 **	40.40 **
Rep (L)	4	0. 26	1.35	12.53	225.52	285.31	0.78
Densities (D)	2	21.93 **	88.74 **	2229.25 **	91,176.13 **	50,563.13 **	58.38 **
L × D	2	1.78 **	6.33 **	331.20 **	3151.63 **	14,971.59 **	54.39 **
Error a	8	0.18	0.52	3.09	192.11	56.68	0.30
Hybrids (H)	27	1.01 **	8.75 **	56.88 **	10,944.20 **	9941.33 **	49.21 **
GCA	7	1.07 **	16.50 **	41.59 **	12,835.71 **	13,527.67 **	67.17 **
SCA	20	0.99 **	6.04 **	62.24 **	10,282.17 **	8686.11 **	42.93 **
H × L	27	1.25 **	18.90 **	21.01 **	2126.28 **	1230.60 **	4.24 **
GCA × L	7	1.56 **	20.27 **	17.14 **	2328.04 **	1621.69 **	5.61 **
SCA × L	20	1.14 **	18.42 **	22.36 **	2055.67 **	1093.72 **	3.75 **
H × D	54	0.20 **	0.98 **	8.77 **	360.55 **	187.93 **	0.71 **
GCA × D	14	0.24	1.45 **	10.49 **	560.46 **	166.93 **	0.73 **
SCA × D	40	0.19	0.82 **	8.17 **	290.58 **	195.28 **	0.70 **
H × D × L	54	1.27 **	13.20 **	16.84 **	621.87 **	1517.64 **	5.45 **
GCA × L × D	14	1.45 **	11.20 **	19.16 **	529.08 **	1951.52 **	6.99 **
SCA × L × D	40	1.21 **	13.89 **	16.02 **	654.34 **	1365.78 **	4.91 **
Error b	324	0.15	0.38	2.41	143.75	45.22	0.25
GCA/SCA		1.08	2.73	0.67	1.25	1.56	1.57

* and ** significant at 0.05 and 0.01 levels of probability, respectively. DTS: days to 50% silking, ASI: anthesis–silking interval, PLHT: plant height, EHT: ear height, LANG: leaf angle, CHLC: chlorophyll content, ED: ear diameter, NRPE: number of rows per ear, NKPR: number of kernels per row, TKW: thousand kernel weight, GYPP: grain yield per plant and GYPH: grain yield per hectare.

**Table 2:** Minimum, maximum and mean values of all the studied traits under three plant densities across two locations.

Trait	Parameter	D1		D2		D3	
		Value	Hybrid	Value	Hybrid	Value	Hybrid
DTS	Minimum	56.17	L_1_ × L_3_	58.50	L_3_ × L_4_	62.52	L_1_ × L_4_
	Maximum	61.50	L_6_ × L_8_	65.00	L_6_ × L_8_	67.60	L_3_ × L_6_
	**Mean**	**58.22**		**61.19**		**64.80**	
ASI	Minimum	2.15	L_2_ × L_5_	2.28	L_2_ × L_5_	3.12	L_2_ × L_5_
	Maximum	4.65	L_3_ × L_7_	5.20	L_3_ × L_7_	5.65	L_3_ × L_7_
	**Mean**	**3.26**		**3.68**		**4.56**	
PLHT (cm)	Minimum	203.17	L_2_ × L_6_	206.00	L_2_ × L_6_	213.35	L_2_ × L_6_
	Maximum	283.00	L_4_ × L_7_	290.63	L_2_ × L_4_	304.35	L_4_ × L_7_
	**Mean**	**240.12**		**249.20**		**263.52**	
EHT (cm)	Minimum	104.32	L_3_ × L_6_	109.49	L_2_ × L_6_	116.42	L_2_ × L_6_
	Maximum	144.14	L_6_ × L_7_	151.23	L_6_ × L_7_	160.29	L_6_ × L_7_
	**Mean**	**117.86**		**125.68**		**133.02**	
LANG (°)	Minimum	25.70	L_4_ × L_5_	24.40	L_4_ × L_5_	22.00	L_4_ × L_5_
	Maximum	45.35	L_3_ × L_7_	42.30	L_3_ × L_7_	39.05	L_3_ × L_7_
	**Mean**	**34.03**		**32.00**		**30.21**	
CHLC (SPAD unit)	Minimum	44.37	L_7_ × L_8_	41.80	L_7_ × L_8_	39.39	L_7_ × L_8_
	Maximum	54.50	L_2_ × L_8_	53.13	L_2_ × L_8_	50.20	L_2_ × L_8_
	**Mean**	**50.34**		**47.59**		**44.23**	
ED (cm)	Minimum	4.70	L_1_ × L_7_	4.20	L_2_ × L_4_	3.60	L_2_ × L_4_
	Maximum	5.80	L_1_ × L_8_	5.15	L_1_ × L_3_	5.00	L_1_ × L_4_
	**Mean**	**5.16**		**4.76**		**4.44**	
NRPE	Minimum	13.00	L_3_ × L_7_	12.30	L_3_ × L_4_	12.18	L_1_ × L_3_
	Maximum	16.40	L_2_ × L_5_	15.20	L_1_ × L_5_	14.70	L_1_ × L_5_
	**Mean**	**14.83**		**13.91**		**13.37**	
NKPR	Minimum	35.20	L_1_ × L_5_	34.00	L_1_ × L_5_	30.29	L_1_ × L_5_
	Maximum	45.10	L_2_ × L_8_	42.00	L_2_ × L_8_	37.95	L_2_ × L_8_
	**Mean**	**40.28**		**37.31**		**33.12**	
TKW (g)	Minimum	315.00	L_3_ × L_8_	291.00	L_5_ × L_6_	276.00	L_5_ × L_7_
	Maximum	405.00	L_2_ × L_8_	374.00	L_1_ × L_4_	353.50	L_1_ × L_4_
	**Mean**	**356.00**		**333.24**		**309.26**	
GYPP (g)	Minimum	130.88	L_3_ × L_7_	122.71	L_3_ × L_8_	103.75	L_3_ × L_7_
	Maximum	236.45	L_2_ × L_8_	215.01	L_2_ × L_8_	187.44	L_2_ × L_8_
	**Mean**	**170.11**		**153.78**		**135.09**	
GYPH ( t ha^−1^)	Minimum	7.79	L_3_ × L_7_	8.76	L_3_ × L_8_	8.78	L_3_ × L_7_
	Maximum	14.07	L_2_ × L_8_	15.35	L_2_ × L_8_	15.61	L_2_ × L_8_
	**Mean**	**10.12**		**10.98**		**11.26**	

**Table 3:** General combining ability (GCA) effects of the eight parental inbred lines for all the studied traits under three plant densities across two locations.

**Inbred Line**	**DTS**			**ASI**			**PLHT**			**EHT**		
	**D1**	**D2**	**D3**	**D1**	**D2**	**D3**	**D1**	**D2**	**D3**	**D1**	**D2**	**D3**
L_1_	−0.84 **	−0.94 **	−1.08 **	−0.27 **	−0.26 **	−0.16 **	−7.22 **	−10.48 **	−3.92 **	10.29 **	8.20 **	5.43 **
L_2_	−0.21	−0.27	0.07	−0.28 **	−0.31 **	−0.30 **	−0.90	−2.58	−7.66 **	−1.35	−1.28	−1.54
L_3_	−0.62 **	−0.81 **	−0.38 **	0.43 **	0.48 **	0.37 **	−3.52 *	−1.12	−4.93 **	−3.74 **	−4.22 **	−3.03 **
L_4_	−0.74 **	−0.60 **	−0.20	−0.05	−0.09	−0.23 **	18.43 **	24.20 **	22.27 **	1.48	3.60 **	4.25 **
L_5_	0.62 **	0.34 *	−0.56 **	−0.31 **	−0.43 **	−0.30 **	−4.29 **	−3.48 *	−2.93 *	−4.21 **	−6.60 **	−6.32 **
L_6_	1.28 **	1.49 **	1.32 **	0.65 **	0.58 **	0.42 **	−8.60 **	−9.06 **	−5.94 **	1.38	1.93	3.43 **
L_7_	0.09	0.30 *	0.95 **	0.02	0.14 *	0.13 *	10.17 **	8.45 **	9.75 **	3.13 **	3.12 **	1.33
L_8_	0.43 **	0.49 **	−0.12	−0.19 **	−0.11 *	0.07	−4.05 **	−5.94 **	−6.64 **	−6.99 **	−4.75 **	−3.55 **
LSD 0.05	0.28			0.11			2.82			2.00		
LSD 0.01	0.36			0.15			3.70			2.63		
**Inbred Line**	**LANG**			**CHLC**			**ED**			**NRPE**		
	**D1**	**D2**	**D3**	**D1**	**D2**	**D3**	**D1**	**D2**	**D3**	**D1**	**D2**	**D3**
L_1_	−2.31 **	−2.55 **	−3.35 **	0.07	0.80 **	2.13 **	0.13 *	0.08	0.20 **	−0.14	0.33 **	0.45 **
L_2_	−1.81 **	−1.62 **	−0.92 **	1.72 **	2.58 **	3.18 **	0.11	−0.08	−0.19 **	0.39 **	0.43 **	0.50 **
L_3_	5.51 **	5.71 **	5.63 **	−1.13 **	−1.32 **	−0.74 **	−0.02	0.13 *	0.06	−0.73 **	−0.55 **	−0.51 **
L_4_	−3.77 **	−2.92 **	−2.87 **	−0.20	−0.42	−1.20 **	0.04	−0.03	−0.06	0.13	−0.29 **	−0.19 *
L_5_	−0.62 *	−0.13	−0.75 **	1.19 **	−0.38	0.16	−0.09	0.02	−0.02	0.42 **	0.27 **	0.16
L_6_	0.84 **	0.55 *	1.34 **	−0.44 *	−0.61 **	−1.81 **	−0.21 **	−0.11	−0.06	0.16	−0.07	−0.08
L_7_	1.74 **	1.18 **	1.15 **	−0.97 **	0.00	−1.34 **	−0.13 *	−0.12 *	−0.07	−0.72 **	−0.52 **	−0.37 **
L_8_	0.42	−0.21	−0.23	−0.24	−0.66 **	−0.38	0.17 **	0.09	0.13 *	0.48 **	0.40 **	0.04
LSD 0.05	0.50			0.43			0.12			0.19		
LSD 0.01	0.66			0.56			0.15			0.25		
**Inbred Line**	**NKPR**			**TKW**			**GYPP**			**GYPH**		
	**D1**	**D2**	**D3**	**D1**	**D2**	**D3**	**D1**	**D2**	**D3**	**D1**	**D2**	**D3**
L_1_	−0.55 *	−0.14	0.58 *	12.63 **	16.08 **	17.98 **	12.19 **	9.92 **	8.79 **	0.73 **	0.71 **	0.73 **
L_2_	1.47 **	1.06 **	1.15 **	18.79 **	16.50 **	10.23 **	19.20 **	19.23 **	15.67 **	1.14 **	1.37 **	1.30 **
L_3_	0.68 **	−0.55 *	−1.17 **	−17.88 **	−10.58 **	−6.60 **	−16.55 **	−16.58 **	−16.95 **	−0.98 **	−1.18 **	−1.39 **
L_4_	−0.62 *	0.08	−0.71 **	6.63 **	3.75 *	1.81	−3.93 **	0.90	−0.58	−0.23 **	0.06	−0.05
L_5_	−1.43 **	−0.73 **	−0.48 *	0.46	−3.08	−5.10 **	6.92 **	4.29 **	4.19 **	0.41 **	0.31 **	0.34 **
L_6_	−0.27	0.46	0.62 *	−5.21 **	−2.58	4.73 *	−13.70 **	−10.97 **	−7.49 **	−0.82 **	−0.78 **	−0.63 **
L_7_	0.56 *	−0.01	0.05	−9.04 **	−13.08 **	−13.27 **	−4.05 **	−4.52 **	−6.16 **	−0.24 **	−0.32 **	−0.50 **
L_8_	0.15	−0.17	−0.05	−6.38 **	−7.00 **	−9.77 **	−0.08	−2.27 *	2.53 *	0.00	−0.16 *	0.21 **
LSD 0.05	0.48			3.68			2.07			0.15		
LSD 0.01	0.63			4.84			2.72			0.20		

* and ** significant at 0.05 and 0.01 levels of probability, respectively. DTS: days to 50% silking, ASI: anthesis–silking interval, PLHT: plant height, EHT: ear height, LANG: leaf angle, CHLC: chlorophyll content, ED: ear diameter, NRPE: number of rows per ear, NKPR: number of kernels per row, TKW: thousand kernel weight, GYPP: grain yield per plant and GYPH: grain yield per hectare.

**Table 4:** Estimates of specific combining ability (SCA) effects of the 28 F_1_ crosses for all the studied traits under the three plant densities across two locations.

**Cross**	**DTS**			**ASI**			**PLHT**			**EHT**			**LANG**			**CHLC**		
	**D1**	**D2**	**D3**	**D1**	**D2**	**D3**	**D1**	**D2**	**D3**	**D1**	**D2**	**D3**	**D1**	**D2**	**D3**	**D1**	**D2**	**D3**
L_1_ × L_2_	0.10	0.36	0.28	0.09	0.19	0.53 **	8.70 **	8.46 **	13.08 **	16.34 **	14.76 **	18.45 **	3.69 **	4.89 **	4.69 **	−1.90 **	−2.68 **	−3.97 **
L_1_ × L_3_	−0.49	−0.42	−0.53	−0.15	0.31 *	0.36 **	2.57	0.49	−7.16 *	7.82 **	11.21 **	9.69 **	3.13 **	3.19 **	3.64 **	0.27	0.95 *	0.44
L_1_ × L_4_	−0.04	−0.58	−1.03 **	0.34 **	0.32 *	0.42 **	−40.63 **	−36.93 **	−29.11 **	6.74 **	1.02	1.82	−1.29 *	−1.61 **	−1.92 **	−1.66 **	−3.95 **	−4.89 **
L_1_ × L_5_	0.96 **	0.68 *	1.41 **	0.04	0.08	−0.10	9.59 **	5.85	19.69 **	9.58 **	9.28 **	6.62 **	−2.74 **	−3.20 **	−3.79 **	0.45	1.01 *	2.35 **
L_1_ × L_6_	−1.05 **	−0.87 **	−1.49 **	−0.10	−0.18	−0.05	6.40 *	7.94 *	3.30	−3.99	−4.10	−6.08 **	−3.21 **	−2.88 **	−1.87 **	0.38	0.84	2.02 **
L_1_ × L_7_	0.53	1.52 **	0.39	−0.55 **	−0.81 **	−0.89 **	23.63 **	21.17 **	2.15	−20.97 **	−18.24 **	−14.06 **	−2.80 **	−2.98 **	−2.32 **	1.84 **	1.75 **	2.58 **
L_1_ × L_8_	−0.01	−0.67 *	0.97 **	0.33 **	0.08	−0.27 *	−10.26 **	−6.98 *	−1.95	−15.52 **	−13.93 **	−16.44 **	3.22 **	2.58 **	1.57 **	0.61	2.09 **	1.46 **
L_2_ × L_3_	1.21 **	2.26 **	1.16 **	0.74 **	0.65 **	0.72 **	22.50 **	34.10 **	35.82 **	5.48 *	3.19	0.98	0.88	0.63	−0.09	−0.65	−1.03 *	−0.57
L_2_ × L_4_	−0.67 *	−0.84 **	−1.30 **	0.43 **	1.06 **	0.88 **	25.00 **	19.90 **	15.73 **	0.82	0.12	−1.07	1.31 *	0.26	−0.60	−0.91	−0.10	0.39
L_2_ × L_5_	1.98 **	−0.44	−0.09	−0.53 **	−0.66 **	−0.83 **	−10.73 **	−10.14 **	−5.97	−3.86	−2.44	2.48	−2.74 **	−1.93 **	−1.52 **	−0.97 *	1.04 *	0.04
L_2_ × L_6_	−0.68*	0.15	0.83 **	0.01	−0.10	−0.03	−27.25 **	−31.46 **	−36.41 **	−12.74 **	−16.48 **	−18.13 **	−0.71	−2.44 **	−1.03	1.66 **	−1.53 **	−0.52
L_2_ × L_7_	−1.10 **	−1.15 **	0.50	−0.30 *	−0.61 **	−0.53 **	−6.69 *	−6.98 *	−9.70 **	−4.10	2.13	−0.21	2.50 **	2.93 **	3.40 **	0.04	0.65	1.38 **
L_2_ × L_8_	−0.84 **	−0.34	−1.38 **	−0.45 **	−0.52 **	−0.75 **	−11.53 **	−13.88 **	−12.56 **	−1.94	−1.28	−2.51	−4.93 **	−4.35 **	−4.86 **	2.73 **	3.64 **	3.25 **
L_3_ × L_4_	1.19 **	−1.21 **	0.34	−0.89 **	−1.30 **	−1.31 **	−15.53 **	−15.19 **	−12.24 **	3.66	3.71	−1.88	−4.06 **	−3.97 **	−2.44 **	3.44 **	5.64 **	6.31 **
L_3_ × L_5_	−1.62 **	−0.65 *	−1.04 **	0.61 **	0.77 **	0.62 **	5.89	3.14	0.11	−3.62	1.51	−0.31	1.89 **	1.74 **	1.24 *	−3.44 **	−2.37 **	−3.38 **
L_3_ × L_6_	−0.27	−1.05 **	1.82 **	−0.87 **	−0.97 **	−0.77 **	5.30	−4.02	0.10	−11.04 **	−10.35 **	−7.78 **	−5.82 **	−3.94 **	−3.81 **	−1.65 **	−3.64 **	−1.92 **
L_3_ × L_7_	1.41 **	1.89 **	−0.01	0.93 **	0.90 **	0.59 **	−18.37 **	−12.94 **	−13.61 **	−0.76	−5.93 **	−7.95 **	4.18 **	3.53 **	2.14 **	2.44 **	2.35 **	0.92
L_3_ × L_8_	−1.43 **	−0.80 **	−1.74 **	−0.37 **	−0.36 **	−0.22	−2.36	−5.59	−3.01	−1.55	−3.34	7.25 **	−0.20	−1.18 *	−0.67	−0.42	−1.89 **	−1.81 **
L_4_ × L_5_	−1.10 **	−0.06	−0.17	−0.47 **	−0.36 **	−0.40 **	−1.56	4.05	9.83 **	−1.54	−3.17	0.54	−3.82 **	−4.42 **	−3.97 **	0.05	−0.77	−1.00 *
L_4_ × L_6_	−0.16	1.74 **	0.64 *	0.02	0.33 *	0.21	16.55 **	24.75 **	13.42 **	−7.77 **	−5.22 *	−2.11	6.61 **	7.69 **	6.35 **	−2.34 **	−0.54	−1.15 *
L_4_ × L_7_	−0.67 *	−0.82 **	−0.88 **	0.34 **	0.01	0.25	14.48 **	7.99 *	8.97 **	−1.93	−0.52	0.89	−2.63 **	−3.24 **	−3.67 **	−0.24	−0.28	−0.59
L_4_ × L_8_	1.44 **	1.79 **	2.39 **	0.23	−0.06	−0.04	1.69	−4.58	−6.59 *	0.03	4.06	1.81	3.88 **	5.28 **	6.25 **	1.66 **	0.01	0.92
L_5_ × L_6_	0.49	0.05	−1.49 **	0.03	0.02	0.11	−5.53	−7.57 *	−11.28 **	−1.55	−0.52	0.62	0.31	0.40	−0.27	2.56 **	2.02 **	2.80 **
L_5_ × L_7_	−0.03	1.39 **	0.68 *	−0.27 *	−0.21	−0.12	−4.32	−5.58	−16.94 **	1.50	−0.83	−5.39 *	3.57 **	3.94 **	5.21 **	0.09	−0.99 *	−0.47
L_5_ × L_8_	−0.67 *	−0.95 **	0.70 *	0.58 **	0.36 **	0.72 **	6.66 *	10.24 **	4.57	−0.51	−3.83	−4.56 *	3.53 **	3.46 **	3.10 **	1.26 **	0.07	−0.33
L_6_ × L_7_	0.0	−1.91 **	−0.01	0.54 **	0.55 **	0.34 **	−9.99 **	−7.05 *	20.23 **	21.93 **	20.87 **	22.87 **	1.75 **	1.39 *	0.63	0.53	1.64 **	−0.78
L_6_ × L_8_	1.67 **	1.90 **	−0.29	0.37 **	0.34 **	0.19	14.52 **	17.40 **	10.65 **	15.17 **	15.80 **	10.60 **	1.07	−0.22	0.02	−1.15 *	1.20 *	−0.45
L_7_ × L_8_	−0.15	−0.91 **	−0.66 *	−0.69 **	0.18	0.36 **	1.26	3.38	8.90 **	4.33	2.52	3.85	−6.57 **	−5.56 **	−5.40 **	−4.70 **	−5.11 **	−3.04 **
LSD 0.05	0.61			0.25			6.23			4.42			1.12			0.95		
LSD 0.01	0.80			0.33			8.19			5.81			1.47			1.24		
**Cross**	**ED**			**NRPE**			**NKPR**			**TKW**			**GYPP**			**GYPH**		
	**D1**	**D2**	**D3**	**D1**	**D2**	**D3**	**D1**	**D2**	**D3**	**D1**	**D2**	**D3**	**D1**	**D2**	**D3**	**D1**	**D2**	**D3**
L_1_ × L_2_	−0.13	−0.17	−0.14	−0.05	−0.16	−0.31	−0.74	−0.47	−1.83 **	13.26 **	6.92	3.06	0.63	−1.34	−2.05	0.04	−0.10	−0.16
L_1_ × L_3_	0.21	0.17	−0.19	−0.69 **	−0.88 **	−1.12 **	−1.34 *	0.43	1.38*	3.93	2.00	−6.11	16.42 **	10.99 **	10.06 **	0.98 **	0.78 **	0.82 **
L_1_ × L_4_	0.32 *	0.19	0.42 **	0.20	0.76 **	0.50 *	0.96	0.97	0.03	27.43 **	21.67 **	24.48 **	−24.25 **	−22.70 **	−18.21 **	−1.44 **	−1.62 **	−1.51 **
L_1_ × L_5_	−0.34 **	−0.07	0.11	0.42 *	0.69 **	0.73 **	−3.04 **	−2.34 **	−1.39 **	−33.40 **	−24.50 **	−16.11 **	2.81	10.32 **	12.43 **	0.17	0.74 **	1.04 **
L_1_ × L_6_	0.03	0.16	0.12	−0.18	−0.36	−0.30	2.61 **	2.77 **	2.93 **	25.26 **	25.00 **	22.06 **	33.52 **	28.56 **	22.11 **	1.99 **	2.04 **	1.85 **
L_1_ × L_7_	−0.45 **	−0.13	−0.26 *	0.25	0.29	0.15	−0.63	−2.06 **	−1.10 *	−9.90 *	−10.50 *	−6.94	−18.98 **	−21.89 **	−18.42 **	−1.13 **	−1.56 **	−1.55 **
L_1_ × L_8_	0.35 **	−0.14	−0.06	0.05	−0.33	0.35	2.19 **	0.70	−0.03	−26.57 **	−20.58 **	−20.44 **	−10.14 **	−3.94	−5.92 *	−0.60 **	−0.28	−0.49 **
L_2_ × L_3_	−0.05	0.08	−0.31*	0.74 **	0.82 **	1.05 **	2.53 **	0.28	−2.66 **	−12.24 **	−7.42	1.14	−15.69 **	−15.14 **	−6.32 **	−0.93 **	−1.08 **	−0.54 **
L_2_ × L_4_	−0.51 **	−0.46 **	−0.59 **	−1.02 **	−0.04	0.02	−2.07 **	−1.56 **	−0.02	−12.74 **	−9.75 *	−14.77 **	−4.15	2.88	8.81 **	−0.25	0.21	0.74 **
L_2_ × L_5_	0.42 **	0.29 *	0.57 **	0.79 **	0.39	0.18	0.00	1.13 *	1.03	18.43 **	22.58 **	14.64 **	25.96 **	23.99 **	18.55 **	1.54 **	1.71 **	1.55 **
L_2_ × L_6_	−0.05	−0.08	0.01	−0.73 **	−0.96 **	−1.18 **	−4.42 **	−3.73 **	−1.99 **	−18.90 **	−14.42 **	−6.69	−33.57 **	−30.75 **	−29.77 **	−2.00 **	−2.20 **	−2.47 **
L_2_ × L_7_	0.36 **	0.43 **	0.63 **	0.33	−0.31	−0.30	1.45 **	0.44	1.60 **	−25.07 **	−25.92 **	−23.69 **	−21.99 **	−25.20 **	−24.60 **	−1.31 **	−1.80 **	−2.07 **
L_2_ × L_8_	−0.04	−0.08	−0.17	−0.05	0.27	0.54 *	3.26 **	3.90 **	3.88 **	37.26 **	28.00 **	26.31 **	48.82 **	45.56 **	35.39 **	2.90 **	3.25 **	2.95 **
L_3_ × L_4_	−0.18	−0.06	0.16	−0.41	−0.76 **	−0.47 *	0.62	−0.34	1.78 **	10.93 **	7.33	−17.94 **	21.41 **	20.99 **	9.83 **	1.27 **	1.50 **	0.80 **
L_3_ × L_5_	−0.05	0.08	0.12	−0.49 *	−0.12	0.19	2.83 **	2.38 **	0.60	27.10 **	15.17 **	22.98 **	−15.18 **	−17.28 **	−14.84 **	−0.90 **	−1.23 **	−1.25 **
L_3_ × L_6_	−0.12	−0.09	0.36 **	1.07 **	0.72 **	0.93 **	−0.33	−0.91	−1.05	−2.24	4.67	5.14	16.68 **	10.35 **	14.54 **	0.99 **	0.74 **	1.20 **
L_3_ × L_7_	−0.01	−0.18	−0.12	−0.36	−0.03	0.11	−2.86 **	−0.35	−0.18	−11.40 **	−10.83 **	−0.86	−17.02 **	1.01	−6.99 **	−1.01 **	0.07	−0.48 **
L_3_ × L_8_	0.19	0.01	−0.02	0.14	0.25	−0.69 **	−1.44 **	−1.49 **	0.12	−16.07 **	−10.92 **	−4.36	−6.61 **	−10.93 **	−6.29 **	−0.39 *	−0.78 **	−0.54 **
L_4_ × L_5_	0.19	0.14	−0.06	−0.04	0.38	0.06	−0.57	−0.66	1.09 *	22.60 **	29.83 **	38.56 **	31.54 **	18.23 **	14.79 **	1.88 **	1.30 **	1.24 **
L_4_ × L_6_	0.22	0.18	0.08	0.21	0.46 *	0.11	0.67	0.15	−2.01 **	3.26	2.33	8.73 *	−9.18 **	−6.02 **	−5.72 *	−0.55 **	−0.43 *	−0.47 **
L_4_ × L_7_	−0.07	−0.16	−0.11	0.78 **	0.21	0.29	1.84 **	1.72 **	0.26	−23.90 **	−22.17 **	−19.27 **	12.90 **	5.74 *	0.74	0.77 **	0.41 *	0.05
L_4_ × L_8_	0.03	0.18	0.09	0.28	−1.01 **	−0.52 *	−1.44 **	−0.27	−1.14 *	−27.57 **	−29.25 **	−19.77 **	−28.26 **	−19.12 **	−10.25 **	−1.68 **	−1.37 **	−0.85 **
L_5_ × L_6_	−0.05	−0.18	−0.36 **	−0.38	−0.91 **	−0.23	1.38 *	0.87	1.30 *	−32.57 **	−35.83 **	−31.86 **	−21.29 **	−10.90 **	−12.19 **	−1.27 **	−0.78 **	−1.01 **
L_5_ × L_7_	0.06	−0.07	−0.14	−0.90 **	−0.86 **	−0.91 **	−1.65 **	−1.07 *	−2.07 **	−2.74	−5.33	−14.36 **	−4.05	0.14	4.58 *	−0.24	0.01	0.36 *
L_5_ × L_8_	−0.24	−0.18	−0.24	0.60 **	0.43 *	−0.02	1.06 *	−0.31	−0.57	0.60	−1.92	−13.86 **	−19.79 **	−24.51 **	−23.32 **	−1.18 **	−1.75 **	−1.94 **
L_6_ × L_7_	0.19	−0.04	−0.31 *	0.46 *	0.69 **	0.49 *	2.79 **	2.35 **	2.28 **	32.93 **	29.17 **	17.81 **	23.51 **	18.01 **	22.66 **	1.40 **	1.29 **	1.87 **
L_6_ × L_8_	−0.21	0.05	0.09	−0.45 *	0.37	0.18	−2.69 **	−1.50 **	−1.47 **	−7.74	−10.92 **	−15.19 **	−9.67 **	−9.25 **	−11.64 **	−0.58 **	−0.66 **	−0.96 **
L_7_ × L_8_	−0.09	0.16	0.31 *	−0.57 **	0.02	0.16	−0.93	−1.03	−0.79	40.10 **	45.58 **	47.31 **	25.64 **	22.19 **	22.03 **	1.53 **	1.58 **	1.82 **
LSD 0.05	0.26			0.42			1.06			8.15			4.57			0.34		
LSD 0.01	0.34			0.55			1.39			10.71			6.01			0.45		

* and ** significant at 0.05 and 0.01 levels of probability, respectively. DTS: days to 50% silking, ASI: anthesis–silking interval, PLHT: plant height, EHT: ear height, LANG: leaf angle, CHLC: chlorophyll content, ED: ear diameter, NRPE: number of rows per ear, NKPR: number of kernels per row, TKW: thousand kernel weight, GYPP: grain yield per plant and GYPH: grain yield per hectare.

**Table 5:** Number of alleles, major allele frequency, gene diversity and polymorphic information content (PIC) of the ten SSR markers used in this study.

Marker	Ch.	Size Range (bp)	No. of Alleles	Major Allele Frequency	Gene Diversity	PIC
phi308707	1	125–140	2	0.63	0.47	0.36
phi96100	2	150–200	2	0.88	0.22	0.19
phi453121	3	150–200	2	0.50	0.50	0.38
phi072	4	100–150	2	0.75	0.38	0.30
phi024	5	100–200	2	0.50	0.50	0.38
umc1014	6	100–150	3	0.50	0.59	0.51
phi112	7	150–200	3	0.50	0.59	0.51
phi015	8	50–150	3	0.50	0.59	0.51
umc1033	9	50–200	6	0.25	0.81	0.79
phi301654	10	100–150	2	0.88	0.22	0.19
Mean			2.7	0.59	0.50	0.41

**Table 6:** Genetic distance (GD) matrix among the eight maize inbred lines based on SSR analysis.

Parent	L_1_	L_2_	L_3_	L_4_	L_5_	L_6_	L_7_	L_8_
L_1_	-	0.43	0.53	0.31	0.71	0.71	0.71	0.78
L_2_		-	0.43	0.53	0.78	0.78	0.71	0.78
L_3_			-	0.43	0.63	0.63	0.63	0.71
L_4_				-	0.63	0.71	0.63	0.71
L_5_					-	0.63	0.43	0.71
L_6_						-	0.43	0.63
L_7_							-	0.53
L_8_								-

**Table 7:** Correlation coefficients among parental genetic distance (GD), F_1_ hybrid performance and SCA for all studied traits across all environments.

Trait	DTS	ASI	PLHT	EHT	LANG	CHLC	ED	NRPE	NKPR	TKW	GYPP	GYPH
r (GD, F_1_)	0.20	−0.26	−0.20	−0.60	−0.09	0.30	0.13	0.26	0.04	−0.21	0.05	0.05
r (GD, SCA)	0.01	−0.26	0.00	−0.55	−0.07	0.29	0.11	0.12	−0.25	−0.26	0.04	0.04
r (F_1_, SCA)	0.69 **	0.78 **	0.75 **	0.83 **	0.70 **	0.80 **	0.85 **	0.71 **	0.90 **	0.83 **	0.80 **	0.80 **

** significant at 0.01 level of probability.

**Table 8:** Code, name and source of the parental maize inbred lines.

Parent Code	Name	Source
L1	IL36	ARC-Egypt
L2	IL94	ARC-Egypt
L3	IL53	ARC-Egypt
L4	IL38	ARC-Egypt
L5	CML538	CIMMYT-Mexico
L6	CML203	CIMMYT-Mexico
L7	CML206	CIMMYT-Mexico
L8	CML441	CIMMYT-Mexico

## Supplementary Materials

The following are available online at https://www.mdpi.com/2223-7747/9/9/1140/s1. Table S1: Mean performance of the 28 F_1_ crosses and the check hybrid SC128 for all the studied traits under the three plant densities across the two locations. Table S2: Physical and chemical soil properties for the two locations during 2018 season. Table S3: List of SSR primers and their sequences used in the present study. Figure S1: Daily maximum temperature (T max), minimum temperature (T min) and solar radiation (SRAD) for the two locations during 2018 season.
