# Supplementary material for: Genetic Diversity and Combining Ability of White Maize Inbred Lines under Different Plant Densities
Source: Plants (Basel). 2020 Sep 3;9(9):1140. doi: 10.3390/plants9091140 (PMC7570016; doi:10.3390/plants9091140)
Supplement: Supplementary file 1 [file plants-09-01140-s001.pdf]

Table S1. Mean performance of the 28 F_1 crosses and the check hybrid SC128 for all the studied traits under the three plant densities across the two locations.

Cross	DTS			ASI			PLHT			EHT			LANG			CHLC		
	D1	D2	D3	D1	D2	D3	D1	D2	D3	D1	D2	D3	D1	D2	D3	D1	D2	D3
$L_1 \times L_2$	57.17	60.27	64.10	2.80	3.29	4.63	240.50	244.50	264.87	143.00	147.00	155.00	33.50	32.60	30.55	50.18	48.27	45.50
$L_1 \times L_3$	56.17	58.95	62.85	3.27	4.22	5.13	231.75	238.00	247.35	132.09	140.50	144.76	40.25	38.23	36.05	49.50	48.00	46.00
$L_1 \times L_4$	56.50	59.00	62.52	3.28	3.65	4.60	210.50	225.90	252.60	136.23	138.13	144.16	26.55	24.80	22.00	48.50	44.00	40.20
$L_1 \times L_5$	58.85	61.20	64.60	2.73	3.07	4.00	238.00	241.00	276.20	133.38	136.20	138.39	28.25	26.00	22.25	52.00	49.00	48.80
$L_1 \times L_6$	57.50	60.80	63.58	3.55	3.83	4.77	230.50	237.50	256.80	125.39	131.35	135.45	29.25	27.00	26.25	50.30	48.60	46.50
$L_1 \times L_7$	57.90	62.00	65.10	2.47	2.75	3.64	266.50	268.25	271.35	110.17	118.39	125.36	30.55	27.53	25.62	51.23	50.13	47.53
$L_1 \times L_8$	57.70	60.00	64.60	3.14	3.39	4.21	218.40	225.70	250.85	105.50	114.84	118.11	35.25	31.70	28.12	50.73	49.80	47.37
$L_2 \times L_3$	58.50	62.30	65.68	4.15	4.50	5.35	258.00	279.50	286.59	118.12	123.00	129.07	38.50	36.60	34.75	50.23	47.80	46.03
$L_2 \times L_4$	56.50	59.42	63.40	3.37	4.33	4.91	282.45	290.63	293.70	118.67	127.75	134.30	29.65	27.60	25.75	50.90	49.63	46.53
$L_2 \times L_5$	60.50	60.75	64.25	2.15	2.28	3.12	224.00	232.91	246.80	108.30	115.00	127.28	28.75	28.20	26.95	52.23	50.80	47.53
$L_2 \times L_6$	58.50	62.48	67.05	3.65	3.85	4.65	203.17	206.00	213.35	105.00	109.49	116.42	32.25	28.37	29.52	53.23	48.00	45.00
$L_2 \times L_7$	56.90	60.00	66.35	2.70	2.90	3.86	242.50	248.00	255.75	115.40	129.29	132.24	36.35	34.37	33.77	51.07	50.80	47.37
L2×L8	57.50	61.00	63.40	2.35	2.74	3.59	223.45	226.70	236.50	107.44	118.00	125.06	27.60	25.70	24.12	54.50	53.13	50.20
L3×L4	57.95	58.50	64.60	2.75	2.78	3.39	239.30	257.00	268.45	119.12	128.40	132.00	31.60	30.70	30.45	52.40	51.47	48.53
L3×L5	56.50	60.00	62.85	4.00	4.50	5.25	238.00	247.65	255.60	106.15	116.00	123.00	40.70	39.20	36.25	46.91	43.50	40.20
L3×L6	58.50	60.75	67.60	3.47	3.78	4.58	233.10	234.90	252.58	104.32	112.67	125.28	34.45	34.20	33.28	47.07	42.00	39.69
L3×L7	59.00	62.50	65.40	4.65	5.20	5.65	228.20	243.50	254.57	116.35	118.28	123.00	45.35	42.30	39.05	50.63	48.60	43.00
L3×L8	56.50	60.00	62.60	3.14	3.70	4.79	230.00	236.45	248.78	105.44	113.00	133.33	39.65	36.20	34.85	48.50	43.70	41.23
L4×L5	56.90	60.80	63.90	2.45	2.80	3.63	252.50	273.88	292.53	113.44	119.14	131.13	25.70	24.40	22.55	51.33	46.00	42.12
L4×L6	58.50	63.75	66.60	3.89	4.50	4.96	266.30	289.00	293.10	112.80	125.63	138.23	37.60	37.20	34.95	47.31	46.00	40.00
L4×L7	56.80	60.00	64.70	3.59	3.74	4.71	283.00	289.75	304.35	120.39	131.50	139.13	29.25	26.90	24.75	48.87	46.87	41.02
L4×L8	59.25	62.80	66.90	3.26	3.42	4.37	256.00	262.79	272.40	112.24	128.22	135.17	34.45	34.03	33.28	51.50	46.50	43.50
L5×L6	60.50	63.00	64.10	3.65	3.85	4.79	221.50	229.00	243.20	113.33	120.13	130.39	34.45	32.70	30.45	53.60	48.60	45.30
L5×L7	58.80	63.15	65.90	2.72	3.17	4.26	241.48	248.50	253.25	118.13	121.00	122.27	38.60	36.87	35.75	50.60	46.20	42.50
L5×L8	58.50	61.00	64.85	3.35	3.50	5.05	238.25	249.92	258.36	106.00	110.13	118.23	37.25	35.00	32.25	52.50	46.60	43.60
L6×L7	59.50	61.00	67.10	4.48	4.95	5.45	231.50	241.45	287.40	144.14	151.23	160.29	38.25	35.00	33.25	49.40	48.60	40.22
L6×L8	61.50	65.00	65.75	4.10	4.50	5.25	241.80	251.50	261.43	127.27	138.30	143.15	36.25	32.00	31.25	48.46	47.50	41.51
L7×L8	58.50	61.00	65.00	2.41	3.89	5.13	247.30	254.99	275.37	118.18	126.20	134.29	29.50	27.30	25.65	44.37	41.80	39.39
Check SC128	61.00	63.00	64.00	3.05	3.68	4.60	245.45	252.00	268.00	122.00	136.00	143.00	37.25	35.40	32.47	52.00	48.20	46.30
LSD 5%		1.02			0.42			10.38			7.36			1.86			1.57	
LSD 1%		1.34			0.55			13.66			9.69			2.44			2.07	

Table S1. Cont.

Cross	ED			NRPE			NKPR			TKW			GYPP			GYPH		
	D1	D2	D3	D1	D2	D3	D1	D2	D3	D1	D2	D3	D1	D2	D3	D1	D2	D3
$L_1 \times L_2$	5.27	4.60	4.30	15.00	14.50	14.00	40.40	37.67	32.87	400.00	372.00	340.00	200.53	180.30	156.25	11.93	12.87	13.02
$L_1 \times L_3$	5.48	5.15	4.50	13.25	12.80	12.18	39.00	36.95	33.76	354.00	340.00	314.00	180.57	156.81	135.75	10.74	11.20	11.31
$L_1 \times L_4$	5.65	5.00	5.00	15.00	14.70	14.13	40.00	38.13	32.87	402.00	374.00	353.00	152.52	140.61	123.85	9.08	10.04	10.32
$L_1 \times L_5$	4.85	4.80	4.73	15.50	15.20	14.70	35.20	34.00	31.67	335.00	321.00	305.50	190.44	177.01	159.25	11.33	12.64	13.27
$L_1 \times L_6$	5.10	4.90	4.70	14.65	13.80	13.43	42.00	40.30	37.10	388.00	371.00	353.50	200.53	180.00	157.25	11.93	12.85	13.10
$L_1 \times L_7$	4.70	4.60	4.30	14.20	14.00	13.60	39.60	35.00	32.50	349.00	325.00	306.50	157.67	136.00	118.05	9.38	9.71	9.83
$L_1 \times L_8$	5.80	4.80	4.70	15.20	14.30	14.20	42.00	37.60	33.47	335.00	321.00	296.50	170.48	156.20	139.25	10.14	11.15	11.60
$L_2 \times L_3$	5.20	4.90	4.00	15.20	14.60	14.40	44.90	38.00	30.29	344.00	331.00	313.50	155.46	140.00	126.25	9.25	10.00	10.52
$L_2 \times L_4$	4.80	4.20	3.60	14.30	14.00	13.70	39.00	36.80	33.40	368.00	343.00	306.00	179.62	175.50	157.75	10.69	12.53	13.14
$L_2 \times L_5$	5.60	5.00	4.80	16.40	15.00	14.20	40.26	38.68	34.67	393.00	368.50	328.50	220.59	200.00	172.25	13.13	14.28	14.35
$L_2 \times L_6$	5.00	4.50	4.20	14.62	13.30	12.60	37.00	35.00	32.75	350.00	332.00	317.00	140.44	130.00	112.25	8.36	9.28	9.35
$L_2 \times L_7$	5.50	5.00	4.80	14.80	13.50	13.20	43.70	38.70	35.77	340.00	310.00	282.00	161.66	142.00	118.75	9.62	10.14	9.89
L2×L8	5.40	4.70	4.20	15.62	15.00	14.45	45.10	42.00	37.95	405.00	370.00	335.50	236.45	215.01	187.44	14.07	15.35	15.61
L3×L4	5.00	4.80	4.60	13.80	12.30	12.20	40.90	36.40	32.87	355.00	333.00	286.00	169.43	157.80	126.16	10.08	11.27	10.51
L3×L5	5.00	5.00	4.60	14.00	13.50	13.20	42.30	38.30	31.92	365.00	334.00	320.00	143.70	122.91	106.25	8.55	8.78	8.85
L3×L6	4.80	4.70	4.80	15.30	14.00	13.70	40.30	36.20	31.37	330.00	324.00	312.00	154.94	135.29	123.95	9.22	9.66	10.33
L3×L7	5.00	4.60	4.30	13.00	12.80	12.60	38.60	36.30	31.67	317.00	298.00	288.00	130.88	132.40	103.75	7.79	9.45	8.78
L3×L8	5.50	5.00	4.60	14.70	14.00	12.20	39.60	35.00	31.87	315.00	304.00	288.00	145.27	122.71	113.14	8.64	8.76	9.42
L4×L5	5.30	4.90	4.30	15.31	14.26	13.39	37.60	35.90	32.87	385.00	363.00	344.00	203.05	175.90	152.25	12.08	12.56	12.68
L4×L6	5.20	4.80	4.40	15.30	14.00	13.20	40.00	37.90	30.87	360.00	336.00	324.00	141.70	136.40	120.05	8.43	9.74	10.00
L4×L7	5.00	4.45	4.20	15.00	13.30	13.10	42.00	39.00	32.57	329.00	301.00	278.00	173.42	154.61	127.85	10.32	11.04	10.65
L4×L8	5.40	5.00	4.60	15.70	13.00	12.70	38.30	36.85	31.07	328.00	300.00	281.00	136.24	132.00	125.55	8.11	9.42	10.46
L5×L6	4.80	4.50	4.00	15.00	13.20	13.21	39.90	37.80	34.40	318.00	291.00	276.50	140.44	134.91	118.36	8.36	9.63	9.86
L5×L7	5.00	4.60	4.20	13.60	12.80	12.25	37.70	35.40	30.47	344.00	311.00	276.00	167.33	152.40	136.45	9.96	10.88	11.37
L5×L8	5.00	4.70	4.30	16.30	15.00	13.54	40.00	36.00	31.87	350.00	320.50	280.00	155.57	130.00	117.25	9.26	9.28	9.77
L6×L7	5.00	4.50	4.00	14.70	14.00	13.40	43.30	40.00	35.93	374.00	346.00	318.00	174.26	155.01	142.85	10.37	11.07	11.90
L6×L8	4.90	4.80	4.60	15.00	14.60	13.50	37.40	36.00	32.07	336.00	312.00	288.50	145.06	130.00	117.25	8.63	9.28	9.77
L7×L8	5.10	4.90	4.80	14.00	13.80	13.20	40.00	36.00	32.19	380.00	358.00	333.00	190.02	167.90	152.25	11.31	11.99	12.68
**Check SC128**	5.30	4.75	4.55	15.50	14.00	13.50	42.00	40.00	37.40	380.00	364.00	326.00	215.00	190.00	170.00	12.79	13.57	14.16
LSD 5%	0.43			0.70			1.76			13.57			7.61			0.56		
LSD 1%	0.57			0.92			2.31			17.86			10.02			0.74		

Table S2. Physical and chemical soil properties for the two locations during 2018 season.

Properties	El-Mahmoudia	Kafrelsheikh
Sand %	16.9	16.56
Silt %	26.5	23.64
Clay %	56.5	59.8
Soil texture	Clay	Clay
Field capacity, %	40.8	41.8
Wilting point, %	20.3	21.2
Bulk density, Mg m^{-3}	1.24	1.38
Organic matter, %	1.85	1.45
$CaCO_3$, %	2.45	4.4
pH*	7.65	8.0
EC **, dS m^{-1}	1.06	1.88
Ca^{++} meq l^{-1}	3.72	13.0
Mg^{++} meq l^{-1}	2.08	12.3
Na^{+} meq l^{-1}	12.8	30.1
K^{+} meq l^{-1}	0.20	0.6
$CO3^{--}$ meq l^{-1}	0.00	0.0
HCO_3^{-} meq l^{-1}	5.4	4.0
CL^{-} meq l^{-1}	8.90	22.7
$SO4^{--}$ meq l^{-1}	4.5	29.3
Available Nitrogen, $mgkg^{-1}$	50.6	30.1
Available phosphorus. $mgkg^{-1}$	16.3	10.0
Available potassium, $mgkg^{-1}$	489.5	650

* pH was determined in soil: water suspension (1:2.5)

** EC was determined in soil paste extract

Table S3. **List of SSR primers and their sequences.**

No.	Marker	Forward primer	Reverse primer
1	phi308707	GCAACAAGATCCAGCCGAT	GTCGCCCTCATATGACCTTC
2	phi96100	AGGAGGACCCCAACTCCTG	TTGCACGAGCCATCGTAT
3	phi453121	ACCTTGCCTGTCCTTCTTTCT	CAAGCAAGACTTTTGATCAGCC
4	phi072	ACCGTGCATGATTAATTTCTCCAGCCTT	GACAGCGCGCAAATGGATTGAACT
5	phi024	ACTGTTCCACCAAACCAAGCCGAGA	AGTAGGGGTTGGGGATCTCCTCC
6	Umc1014	GAAAGTCGATCGAGAGACCCTG	CCCTCTCTTCACCCCTTCCTT
7	phi112	TGCCCTGCAGGTTCACATTGAGT	AGGAGTACGCTTGGATGCTCTTC
8	phi015	GCAACGTACCGTACCTTTCCGA	ACGCTGCATTCAATTACCGGGAAG
9	umc1033	CTTCTTCGTAAAGGCATTTTGTGC	GTGCGGGATTCCTTAGTTTGC
10	phi301654	GAATGCATGCTTTTCAAGGAC	CGCACAGAGAGCAGAACG

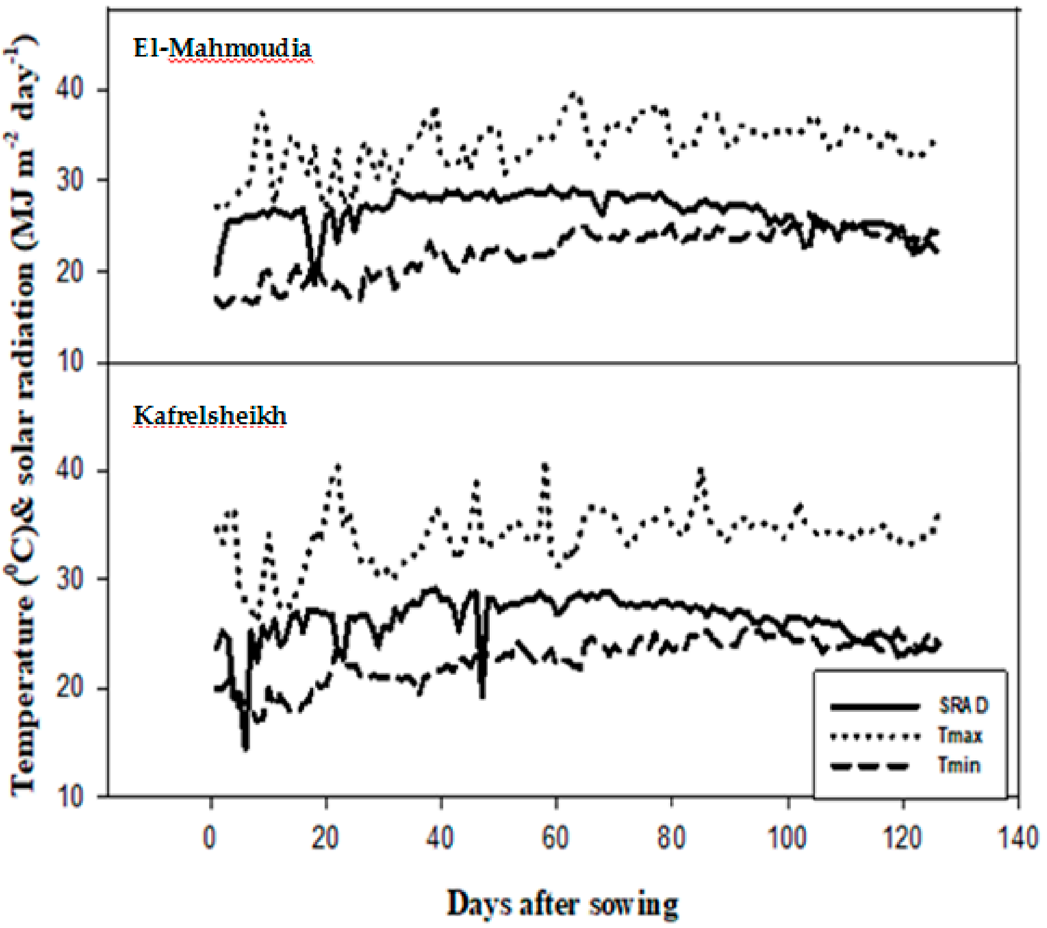

Figure S1. Daily maximum temperature (T max), minimum temperature (T min) and solar radiation (SRAD) for the two locations.
